# P189: Development of on line continuous education programme in a tertiary care hospital of a developing country

**DOI:** 10.1186/2047-2994-2-S1-P189

**Published:** 2013-06-20

**Authors:** S Singh

**Affiliations:** 1 Infection Control, Amrita Institute of Medical Sciences, Kochi, India

## Summary

Training is an essential component of HAIs, wher IT can play a major role. The objective was to develop an e learning continuous education programme for knowledge and skill based competency building in infection control. An interview was conducted and an on line e learning module was developed by a team. It improved the participation of HCWs in training. The competency, knowledge, practices and updating of recent advances was impressive. Process, outcome indicators improved and it proved to be cost effective.

## Introduction

Training with IT aid would be the call of the day.

## Objectives

Develop an e learning continuous education programme in infection control.

## Methods

An interview was conducted to understand the impact of continuous education class room sessions, Pilot study was conducted. Team was included towards programing and deveopment. Every staff will get a user id and a password. An article with 10 MCQs, was released by the in-service administrator, open for the staff for the period of two weeks. They can attempt MCQs and grade them simultaneously and do simlulation training.

## Results

1344 nursing staff, 48% of the staff were able to attend the class room session. 38% of the lectures were missed. Important topics were missed by the staff. Skill based learning was appreciated more. 88% of staff felt that e-learning improved their knowledge, skills and practice. 76% felt it allows flexibility in attending the sessions. 91% of the staff have started attending. Quality indicators in infection control and nursing, staff’s knowledge and skills had improved from 43% to 87%. Hand washing compliance improved from 47% to 78% in the hospital after institution of e learning and continuous reminders. Cost towards training has reduced from Rs 7 lacs per annum to 1.5 lacs per annum.

## Conclusion

There is a remarkable improvement in knowledge, competency and skills. Attendance, process, outcome and cost showed improvement.

## Disclosure of interest

None declared

